# High Titer Persistent Neutralizing Antibodies Induced by TSST-1 Variant Vaccine Against Toxic Shock Cytokine Storm

**DOI:** 10.3390/toxins12100640

**Published:** 2020-10-02

**Authors:** Andreas Roetzer, Norbert Stich, Nina Model, Michael Schwameis, Christa Firbas, Bernd Jilma, Martha M. Eibl

**Affiliations:** 1 Biomedizinische Forschung & Bio-Produkte AG, Lazarettgasse 19, 1090 Vienna, Austria; andreas.roetzer@biomed-research.at (A.R.); norbert.stich@biomed-research.at (N.S.); nina.model@biomed-research.at (N.M.); 2 Department of Clinical Pharmacology, Medical University of Vienna, 1090 Vienna, Austria; michael.schwameis@meduniwien.ac.at (M.S.); christa.firbas@meduniwien.ac.at (C.F.); bernd.jilma@meduniwien.ac.at (B.J.); 3 Department of Emergency Medicine, Medical University of Vienna, 1090 Vienna, Austria

**Keywords:** Staphylococcus, toxic shock syndrome, neutralizing antibodies, immunogenicity

## Abstract

Staphylococcal superantigen toxins lead to a devastating cytokine storm resulting in shock and multi-organ failure. We have previously assessed the safety and immunogenicity of a recombinant toxic shock syndrome toxin 1 variant vaccine (rTSST-1v) in clinical trials (NCT02971670 and NCT02340338). The current study assessed neutralizing antibody titers after repeated vaccination with escalating doses of rTSST-1v. At study entry, 23 out of 34 subjects (67.6%) had neutralizing antibody titers inhibiting T cell activation as determined by ^3^H-thymidine incorporation at a serum dilution of ≤1:100 with similar figures for inhibition of IL-2 activation (19 of 34 subjects, 55.9%) as assessed by quantitative PCR. After the first vaccination, numbers of subjects with neutralization titers inhibiting T cell activation (61.7% ≥ 1:1000) and inhibiting IL-2 gene induction (88.2% ≥ 1:1000) increased. The immune response was augmented after the second vaccination (inhibiting T cell activation: 78.8% ≥ 1:1000; inhibiting IL-2 induction: 93.9% ≥ 1:1000) corroborated with a third immunization months later in a small subgroup of subjects. Assessment of IFNγ, TNFα and IL-6 inhibition revealed similar results, whereas neutralization titers did not change in placebo participants. Antibody titer studies show that vaccination with rTSST-1v in subjects with no/low neutralizing antibodies can rapidly induce high titer neutralizing antibodies persisting over months.

## 1. Introduction

About 10% to 20% of hospital or community acquired infections are caused by the Gram-positive commensal *Staphylococcus aureus*, which are important players in bacteremia and the leading cause of bacterial endocarditis [1,2]. Virulence factors of *S. aureus*, e.g., exotoxins, cause significant diseases, including pneumonia, infective endocarditis, osteomyelitis, and toxic shock syndrome (TSS) [3]. Fatal systemic diseases, e.g., TSS, have high mortality rates known to be caused by superantigen toxins [4,5]. These toxins may be produced and secreted by both MSSA and MRSA [6]. They trigger exaggerated T cell activation and dysregulated inflammation leading to a cytokine storm [7,8].

Experimental application of superantigens leads to a devastating inflammatory response with dysregulated release of cytokines resulting in shock and multi-organ failure [9,10,11]. In most reports, consequences of uncontrolled T cell activation and release of inflammatory cytokines are taken as a single entity. Inflammatory cytokines, unlike mediators of T cell activation, are produced by different cells of the immune system. The question whether T cell activation plays a pivotal role in the initiation and maintenance of inflammation is still not clarified.

Among exotoxins of *S. aureus*, TSST-1 belongs to the pyrogenic toxin class of superantigens and is a causative agent in toxic shock syndrome and septic shock [12,13]. TSST-1 is the main cause of menstrual-associated TSS and is involved in 50% of non-menstrual TSS, besides superantigens B and C [14,15,16].

Neutralizing antibodies are known to be of great importance in the control of toxin-mediated diseases. Given the increasing antibiotic resistance of staphylococci, the development of vaccines is of great priority [17]. Most of the vaccine candidates tested in clinical studies have been aimed at preventing or treating infection, and mainly contained antigens expressed on the bacterial surface [18,19]. These approaches tested in clinical trials have not been successful so far.

Our hypothesis is that staphylococcal exotoxins such as superantigens and cytolytic toxins play a pivotal role in the pathogenesis of systemic disease, e.g., TSS and septic shock. Toxin-neutralizing antibodies are protective and prevent toxin-mediated disease, e.g., tetanus and diphtheria [20]. The recombinant detoxified toxic shock syndrome toxin 1 variant vaccine (rTSST-1v) is the first component of our multi-component candidate vaccine to prevent systemic menstruation and sepsis-associated TSS. Its safety, tolerability (primary objective) and immunogenicity (secondary objective) have previously been assessed in a randomized, double-blind, adjuvant-controlled, dose escalation first-in-man clinical trial [21]. rTSST-1v was safe and well-tolerated and induced a strong anti-TSST-1 binding antibody response after two immunizations.

We invited participants of this first-in-man trial to take part in subsequent single-blinded observational studies to investigate kinetics of neutralizing antibody titers and the immunological response to a third vaccination, which was also well tolerated. All participants had high binding antibody titers 15 months after the second immunization and stable high level of binding titers throughout. The third vaccination resulted in a further increase in binding antibody titers.

Reports on immunological assessment of functional antibodies in the early phase of clinical trials are still scarce. The current study assessed neutralizing antibody titers in 33 (out of 46) participants immunized with rTSST-1v, where all 33 participants were analyzed as a single group, irrespective of the dose of the vaccine. Twelve (out of 46) participants received placebo (Al(OH)_3_ adjuvant). Here, we present a detailed description of toxin-neutralizing antibodies: inhibition of exaggerated T-cell activation and neutralization of induction of gene expression of inflammatory cytokines.

## 2. Results

One aim of this study was to assess the neutralization antibody titers after intramuscular administration of the BioMed rTSST-1v in healthy adults. In this adjuvant-controlled study, dose escalation of TSST-1v was performed starting at the dose of 100 ng. As the upper dose limit of 30 µg was not considered dose-limiting in terms of safety, immunizations were repeated at the same dose level 1–2 months later. After each immunization, the specific immune response was repeatedly monitored by evaluation of rTSST-1 antibodies. Binding antibody titers of this first-in-man study were described in detail in our previous work [21]. It also included a brief analysis of T cell activation at the end of the study after the second immunization.

**Figure 1:**
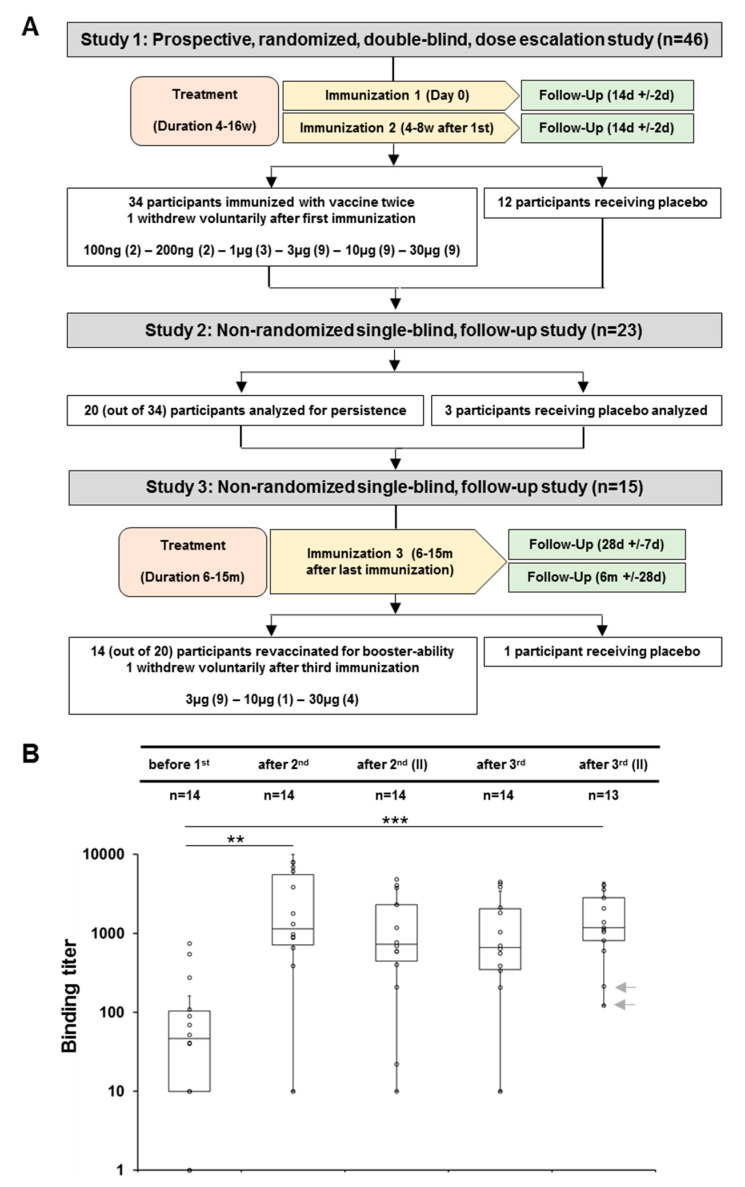
(**A**) Disposition of subjects. Trial flow chart of first-in-man and follow-up studies. Time tables indicate time periods of vaccination. At each time point sera were taken for binding and neutralizing antibody titer assessment. Vaccine doses are given for Study 1 and Study 3, number or participants per group are added in parentheses. (**B**) Persistence and booster-ability of antisera from invited participants of a follow-up study. Increase of binding titers in a log-lin scale of invited participants, who received a third immunization. Out of fifteen participants, who followed the invitation, fourteen were included in the per-protocol evaluation. One participant with a 100-fold increase 28 days after the booster decided to withdraw before the last time point. Additional sera were taken before (designated as after 2nd (II)), 28 days (after 3rd) and 6 months (after 3rd (II)) after the third immunization. *p*-values were calculated in a paired, two-tailed t-test (** *p* < 0.01, *** *p* < 0.001). Two grey arrows in the fifth column indicate the binding titers of the two non-responders finally displaying seroconversion.

Here, neutralization of sera was analyzed in 45 subjects in detail (Figure 1A). One subject was excluded from the per protocol population after voluntary subject withdrawal, leaving 33 subjects with dose escalation of rTSST1v and 12 placebo recipients. These 45 subjects were then invited to participate in the second part of the study to evaluate long-term neutralizing antibody levels of rTSST-1v. From 23 responding subjects, 15 proceeded to the final part of the study, whereas eight subjects were not interested to continue. These 15 subjects were included in the analysis subpopulation, although one subject was a drop-out at the last study visit.

The assessment of binding antibodies against the rTSST-1v vaccine candidate as secondary objective of the clinical trial demonstrated seroconversion [21]. Further detailed analysis of binding titers of the fourteen subjects immunized in the final follow-up part revealed an immediate increase and stable high level of binding titers throughout both studies (Figure 1B). The unchanging increase of binding titers lasting until the end of the follow-up study was statistically and highly significant. The subjects with boostered seroconversion also included two previous non-responders (indicated by arrows).

The results presented here combine all administered dose levels as even participants who received 100 ng showed a vigorous antibody response. As read-out for uncontrolled T cell activation, ^3^H thymidine incorporation during lymphocyte proliferation and measurement of gene induction of IL-2 and IFNγ were carried out and inhibition by serum dilutions determined. In addition, gene induction of inflammation cytokines TNFα and IL-6 was assessed. We compare the results of vaccinated and placebo participants. Early clinical studies usually report on binding antibodies (ELISA), whereas presentation of neutralizing antibodies in early studies is rare.

### 2.1. A Rapid Increase of Neutralization Antibody Titer Levels after the First Immunization

**Figure 2:**
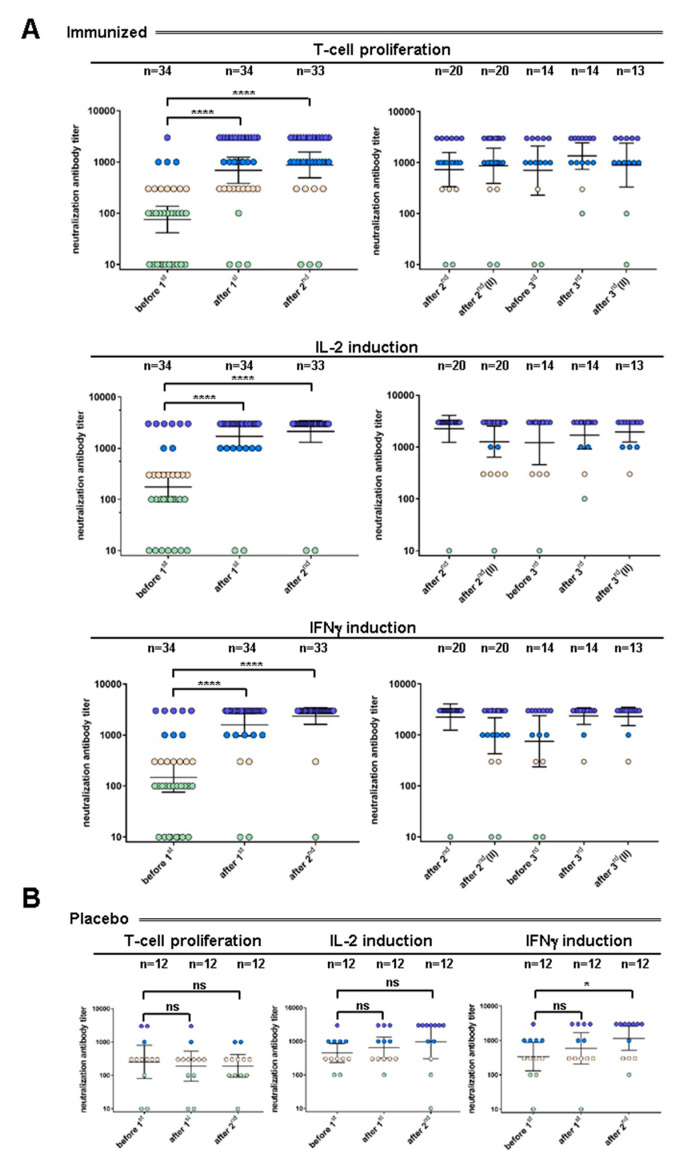
Neutralization of T cell activation and cytokine induction by sera from study participants. Scattered dot plots with geometric means (including 95% CI) of neutralizing antibody titers of sera from immunized (**A**) and placebo (**B**) subjects throughout all time points (serum dilutions ≤100 in green, 300 in pink, 1000 in blue, 3000 in violet). Thirty-four subjects receiving the rTSST-1v and 12 placebo recipients (Al(OH)_3_) were analyzed. One subject was a drop-out after the first vaccination. Twenty subjects were analyzed for persistence of neutralization titers. Fourteen subjects proceeded to the final part of the study, and one subject was a drop-out at the last study visit. Neutralizing sera from all vaccinated subjects (*n* = 33) are shown from before the first immunization (day 0/before 1st), after the first immunization (14 days / after 1st), twice after the second immunization (14 days (after 2nd) and 6 to 15 months (after 2nd II)). In the follow-up study, the schedule was set for 6 to 15 months after the second immunization (before 3rd), the mean elapse time being 294 days. Further values of titers were determined 28 days after the third immunization and six months after the third immunization (after 3rd II). *p* values were calculated by the paired, two-tailed Wilcoxon test (**** *p* < 0.0001, * *p* < 0.05). Geometric mean titers including 95% CI of neutralizing antibodies before the first and 14 days after the second immunization in immunized and placebo recipients determined by inhibition of T cell proliferation and cytokine gene induction are shown.

At study entry, 23 out of 34 subjects (67.6%) had serum antibody titer dilutions ≤1:100 inhibiting T cell activation (Figure 2A, upper panel). Among them, 12 (52.2%) had no measurable antibody titers at study entry and 11 subjects (47.8%) had serum dilutions of 1:100 for neutralizing antibody titers. Out of the 34 subjects, 11 individuals had higher serum antibody titer dilutions than 1:100 for neutralizing T cell proliferation. In this heterogeneous group, dilutions of serum titers varied between 1:300 and 1:3000. The individual having neutralizing antibodies at a serum dilution of 1:3000 might have had an unrecognized previous subclinical infection, e.g., small cuts in the nasal mucosa.

Fourteen days after the first i.m. administration of rTSST-1v, numbers of subjects with neutralization titers increased. Independent of the administered dose of rTSST-1v, 21 out of 34 (61.7%) subjects had high neutralizing antibody titers (serum dilution ≥ 1:1000) inhibiting T cell activation, with 13 subjects (38.2%) having already a serum dilution of 1:3000. Only four subjects (11.8%) still had an antibody titer dilution of ≤1:100. Of the 23 subjects having a ≤1:100 serum dilution of neutralizing antibody titers at study entry, 19 showed seroconversion already after the first immunization (82.6%). Geometric means of neutralization titers for T cell proliferation increased 9.1-fold from 75.3 (95% CI: 41.4–136.9) to 688.9 (95% CI: 383.6–1237) after the first immunization. 

Analysis of inhibition of T cell associated cytokines revealed similar results (see Figure 2A middle and lower panel): at study entry, 19 out of 34 subjects (55.9%) had serum dilutions ≤1:100 for neutralizing antibodies inhibiting IL-2 gene activation, and 17 out of 34 subjects (50.0%) had serum dilutions ≤1:100 for neutralizing antibodies inhibiting IFNγ gene activation. Fourteen days after the first i.m. administration of rTSST-1v, 30 out of 34 participants (88.2%) had serum dilutions ≥1:1000 for neutralizing antibodies inhibiting IL-2 gene induction, and 32 out of 34 subjects (94.1%) had serum dilutions ≥1:1000 for neutralizing antibodies inhibiting IFNγ gene activation. Neutralization titers for all assays increased significantly 14 days after the first administration of TSST-1v (*p* < 0.0001).

### 2.2. Manifestation of High Titers of Neutralizing TSST-1 Antibodies after Two Immunizations

Fourteen days after the second i.m. administration of rTSST-1v, neutralizing antibody titers further increased. A total of 78.8% of subjects had serum dilutions ≥1:1000 for neutralizing antibody titers inhibiting T-cell activation, and 93.9% of subjects had serum dilutions ≥1:1000 for neutralizing antibody titers inhibiting IL-2 and IFNγ gene induction. Concerning T cell proliferation, the sera from three participants did not respond after two rounds of immunization. The read outs for IL-2 and IFNγ gene activation were comparable; one participant (IL-2) and two participants (IFNγ) showed no response after two rounds of immunization.

In the second part of this study, 23 subjects were evaluated for long-term persistence of antibody titers 6–15 months after their last immunization; twenty subjects had received rTSST-1v twice, and 3 recipients had received placebo. Neutralizing antibody titers remained high in all assays tested (Figure 2, right panels). Sixteen out of 20 (80.0%) had serum dilutions ≥1:1000 for neutralizing antibodies inhibiting T cell proliferation, 15 out of 20 (75.0%) had serum dilutions ≥1:1000 for neutralizing antibodies inhibiting IL-2 gene induction, and 16 out of 20 (80.0%) had serum dilutions ≥1:1000 for neutralizing antibodies inhibiting IFNγ gene induction.

Neutralization titers for T cell activation did not change in placebo participants (Figure 2B). Neutralizing antibody titers were significantly higher (*p* < 0.0001) in the rTSST-1v vaccinated cohort compared with in the placebo group 14 days after the first and 14 days after the second immunization. As expected from the results of the first part of the study, none of the three subjects receiving placebo, which were analyzed for persistence of antibody titers, presented with a neutralization titer classified as seroconversion (data not shown).

### 2.3. Neutralization Antibody Titer Levels for Inflammatory Cytokine Gene Production

**Figure 3:**
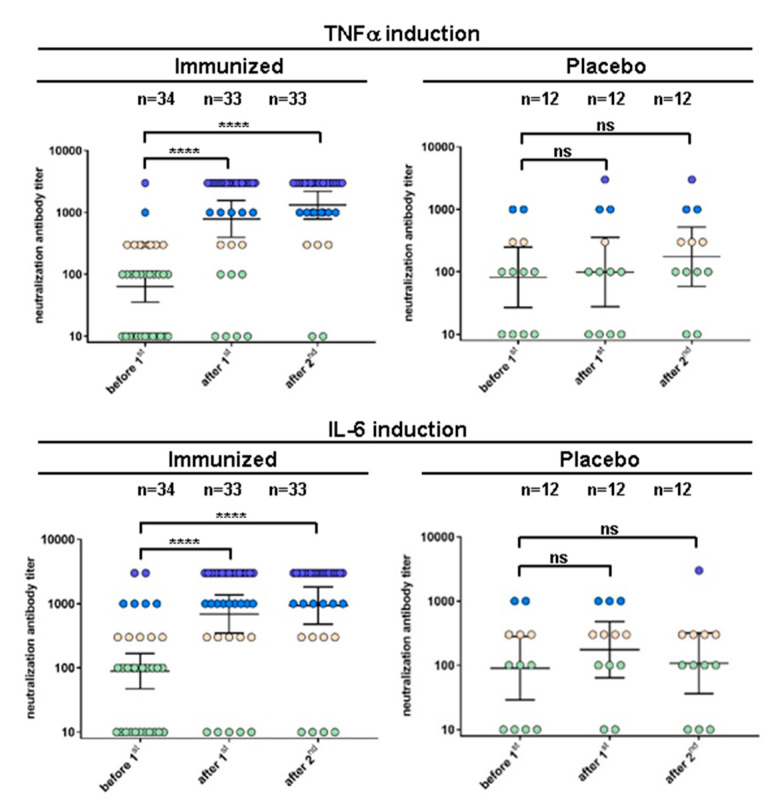
Neutralization of inflammatory cytokine induction by sera from study participants. Scattered dot plots with geometric means (including 95% CI) of neutralizing antibody titers of sera from immunized and placebo subjects (serum dilutions ≤100 in green, 300 in pink, 1000 in blue, 3000 in violet). Thirty-four subjects receiving the rTSST-1v and 12 placebo recipients (Al(OH)_3_) were analyzed. One subject was a drop-out after the first vaccination. Neutralizing sera from all vaccinated subjects (*n* = 33) are shown from before the first immunization (day 0/before 1st), after the first immunization (14 days / after 1st) and twice after the second immunization (14 days/after 2nd). *P* values were calculated by the paired, two-tailed Wilcoxon test (**** *p* < 0.0001). Geometric mean titers including 95% CI of neutralizing antibodies before the first and 14 days after the second immunization in immunized and placebo recipients determined by inhibition of T cell proliferation and cytokine gene induction are shown.

As read-out for inhibition of inflammatory cytokine gene induction, expression rates of the two prominent cytokines TNFα and IL-6 were determined. The increase of neutralizing antibody titers (Figure 3) was assessed as for T cell activation associated cytokines. Twenty-three out of 34 subjects (67.6%) had serum dilutions ≤1:100 for neutralizing antibody titers inhibiting TNFα gene activation at entry, and 22 out of 34 subjects (64.7%) had serum dilutions ≤1:100 for neutralizing antibodies inhibiting IL-6 gene induction.

After the first i.m. administration of rTSST-1v, 24 (70.6%) out of 34 vaccinated subjects had serum dilutions ≥1:1000 for neutralizing antibodies inhibiting TNFα and Il-6 gene induction. Fourteen days after the second administration of rTSST-1v, the neutralizing antibody titers further increased, with high neutralizing titers in 84.8% of subjects inhibiting TNFα gene induction and 75.8% of subjects inhibiting IL-6 gene induction (serum dilution ≥1:1000). All titer increases were statistically and highly significant, indicating robust results even when including subjects with low immune response and low doses of rTSST-1v.

### 2.4. TSST-1 Neutralizing Antibody Titers Remained High after Three Immunizations

From 15 subjects proceeding to the final part of the study, 14 subjects received a third vaccination with a mean elapse time of 294 days after the second immunization in an exploratory setting. One recipient received placebo. One subject withdrew voluntarily before the last blood sampling. The majority of the 14 participants scheduled to receive a third vaccination with rTSST-1v still had high neutralization titers for T cell activation (serum dilution ≥1:1000) before the third immunization (*n* = 11, 78.5%). Ten out of 14 (71.4%) had ≥1:1000 antibody serum dilutions inhibiting both IL-2 gene induction and IFNγ gene induction (see Figure 2, right panels).

Twenty-eight days after the third vaccination, neutralizing antibody titers remained high: 12 out of 14 subjects (85.7%) had ≥1:1000 antibody serum dilutions inhibiting T cell activation, and 2 subjects had low or no antibody titers (≤1:300). Twelve out of 14 subjects (85.7%) had ≥1:1000 antibody titer serum dilutions inhibiting IL-2 gene induction, and 13 out of 14 subjects (92.8%) had ≥1:1000 antibody serum dilutions inhibiting IFNγ gene induction.

Six months after the third immunization, the neutralizing antibody titer for T cell proliferation was still significantly increased (*p* = 0.001). After six months, geometric means of antibody titers for T cell activation, IL-2 gene induction and IFNγ gene induction approached the levels observed 14 days after the second immunization.

## 3. Discussion

Induction of neutralizing antibodies is common practice in the prevention and treatment of toxin-mediated diseases. Our hypothesis was that staphylococcal exotoxins, superantigen toxins and cytolysins are the major culprits in systemic disease caused by staphylococci. *S. aureus* is able to produce a number of superantigen toxins, depending on its genetic background [22,23]. Their release is key for dysregulated activation of immunity due to uncontrolled production of T cell and inflammatory cytokines [24,25].

Our first main target was the superantigen toxin TSST-1: rTSST-1v was our first vaccine candidate for prevention and treatment of toxic shock syndrome. Our detoxified double-mutant rTSST-1v under investigation was extensively tested in vitro in human blood mononuclear cells, and in vivo in rabbits and mice [26,27].

Three rounds of immunization have shown that rTSST-1v is well tolerated and immunogenic, as far as binding antibodies are concerned, and that neutralizing antibodies are produced which might be protective. Several previous studies suggest that approximately 30% of the population at large is colonized with *S. aureus* [28]. This could explain why about 30% of the subjects in our studies already had antibodies to TSST-1 at baseline.

In the present study, we analyzed neutralizing antibodies against T cell activation and inflammatory cytokine secretion in the 46 enrolled participants. Thirty-three participants received escalating doses of the vaccine between 100 ng–30 µg, but results will be discussed irrespectively of the dose.

At study entry, the geometric mean of inhibition of ^3^H thymidine incorporation in the participants was 75.3 and increased by one order of magnitude after the first immunization, which also applied to neutralizing antibodies for T cell cytokines and inflammatory cytokines. Twenty-three (two-thirds) of the subjects had no (*n* = 12) or very low (1:100, *n* = 11) neutralizing antibodies at entry (prior to the first immunization), as determined by inhibition of ^3^H incorporation. Despite a different fold increase, these two groups showed a comparable titer increase for T cell activation: 7 out of 12 (no titer) and 6 out of 11 (1:100) subjects had a serum titer ≥1:1000 after the first round of immunization. However, the whole group with a titer dilution ≤1:100 was not homogenous: two participants had no antibodies throughout the study. Overall, the majority of these subjects (*n* = 13) had an increase to 1:1000 and higher as soon as 14 days after vaccination, suggesting prior contact with the antigen.

The subjects who had high binding and neutralizing antibodies at entry had increases in antibody titer about two-fold, but they did not reach the pre-defined four-fold increase for seroconversion. Therefore, they were considered non-responders in the per protocol analysis [21]. These findings may be considered in the design of future vaccine studies because individuals with neutralizing antibodies might have been protected before entry. Thus, one may consider to use high titer neutralizing antibodies in addition to seroconversion (four-fold increase in binding antibody titer) as a parameter for the evaluation of potency. These findings are important for the planning of clinical vaccine studies, such as in cases of elective surgical interventions.

We were surprised to see such a rapid increase after the first immunization in individuals who had no or very low antibodies because this pattern suggests a secondary response. This could be due to previous exposure to very low amounts of antigen with great time intervals before study entry and/or to the possibility that the first contact was with very low amounts of the toxin produced during temporary colonization. However, it cannot be excluded that the kinetics of the immune response between a superantigen and a normal antigen might differ. As we did not observe comparable kinetics in animal experiments (rabbits and mice), we thus prefer the explanation of primary contacts with very low doses of the respective antigen.

Furthermore, antibody kinetics at study entry and after the first and second round of immunizations indicated that subjects might have mounted a primary response after the first contact with the antigen. The group of 23 subjects who were negative for neutralizing antibodies at entry included individuals who responded with significantly high titers after the second immunization suggestive for a primary response. After the first and second immunization, the kinetics of IL-2 and IFNγ gene induction paralleled T cell activation assessed by ^3^H incorporation. After the third immunization, the response of inflammatory cytokines was more diverse. This vigorous increase in the immune response was absent in placebo participants.

Reports from other studies suggested that the induction of high titer antibodies might be a better indicator of protection than other parameters used for evaluation [29,30]. With regard to staphylococcal superantigen toxins, specific antibodies inhibiting T-cell activation are of great importance. When evaluating a possible protective effect, the dysregulated cytokine gene activation/ secretion needs to be considered because the uncontrolled inflammation drives the clinical symptomatology, with possible severe sequelae in the course of the disease in terms of morbidity and mortality.

In conclusion, the results of the antibody titer studies show that vaccination with a non-toxic rTSST-1 variant vaccine in subjects with no/low neutralizing antibodies can induce high titers of neutralizing antibodies. A diagnostic vaccination might clarify the status of responder or no responder in as little as two to four weeks. The rapid induction of high titer neutralizing antibodies is an important parameter in addition to seroconversion for the evaluation of protectivity in vaccine studies of toxin-mediated diseases, but also in other conditions. A stable, high neutralizing antibody titer over an extended period of time—months in our study—would be an excellent tool for disease prevention in intensive care units and for patients with elective surgery (e.g., heart, orthopedic, plastic, etc.) operations.

## 4. Materials and Methods

### 4.1. Study Design

Study 1 was a prospective, randomized, dose-escalation study of the rTSST-1 Variant Vaccine. Forty-six participants were randomly assigned to one of six dose-escalation groups (100 ng, 300 ng, 1 µg, 3 µg, 10 µg and 30 µg; *n* = 34) of rTSST-1v, the number of subjects per group increasing with dose. Each group included placebo recipients receiving the adjuvant Al(OH)_3_ (*n* = 12) only. Forty-five participants received a second vaccination four weeks later (one dropped out after first immunization). The primary endpoint was safety and tolerability of r-TSST-1v, and the secondary endpoint was immunogenicity as determined by TSST-1 binding antibody titers. The candidate vaccine showed a good safety profile and high titers of binding antibodies [21].

Study 2: Participants in study 1 were invited for determination of TSST-1 antibody persistence 6–15 months after the second immunization; 23 participants returned for antibody determination.

Study 3: 15 (out of 23) participants volunteering for a third immunization in an exploratory setting received a booster immunization with the respective prior dose (one received placebo) at the time they presented for TSST-1 antibody determination in study 2. Subjects who had received 3 µg of vaccine or less received 3 µg of vaccine. Vaccinees were followed up for antibody determination for six months after vaccination (see Figure 1).

Neutralizing antibodies to TSS toxin after each vaccination with rTSST-1v were determined using samples from all three studies, which were stored for exploratory purposes.

The clinical and laboratory safety parts of the studies were conducted at the Department of Clinical Pharmacology at the Medical University of Vienna, 1090 Vienna, Austria (Prof. Jilma, M.D., PI). The studies for immunogenicity and exploratory endpoints, such as neutralizing antibodies, were performed by the Laboratory of the Biomedizinische Forschung & Bio-Produkte AG, 1090 Vienna, Austria, with study director Martha M. Eibl, M.D. They were approved by the Ethics Committee of the Medical University of Vienna 1090 Vienna, Austria (European Council number 1655/2013), and performed in compliance with the Declaration of Helsinki, Good Clinical Practice guidelines, and the Note for Guidance on Clinical Evaluation of Vaccines. The studies were registered with EudraCT (2013-003716-50) and clinicaltrials.gov (NCT02971670 and NCT02340338). Ethical approval code (EK Nr): 2046/2013; approval date: 21 January 2014.

All participants were enrolled from the Department of Clinical Pharmacology at the Medical University of Vienna, 1090 Vienna, Austria. Healthy adults aged 18–64 years were eligible for inclusion in studies. Oral and written informed consent was obtained before any study-related activities began. 

### 4.2. Peripheral Blood Mononuclear Cell (PBMC) Isolation

PBMCs were isolated from fresh heparinized blood of healthy adult donors through density gradient centrifugation using Lymphoprep (Axis-Shield PoC, Oslo, Norway), as already described [31]. Cells were cultured in complete medium (RPMI 1640, Gibco, Grand Island, NY, USA) with stable glutamine (GE Healthcare, Little Chalfont, UK), 10% fetal calf serum (HyClone, Logan, UK), 100 U/mL penicillin and 100 µg/mL streptomycin (Invitrogen, Carlsbad, CA, USA). Cells were used for the exploration of T-cell proliferation and cytokine production of immune cells.

### 4.3. PBMC Proliferation (^3^H Thymidine Incorporation)

For ^3^H thymidine incorporation, freshly isolated human peripheral blood mononuclear cells (MNC; 1 × 10^6^/mL cells) were cultured in flat-bottom tissue culture plates (Sarstedt, Newton, MA, USA) and stimulated with 0.1 ng/mL wild-type recombinant TSST-1 (toxin) for four days in a humidified atmosphere. Negative serum was taken from a human unvaccinated donor, which was not included in the study. Phytohemagglutinin was used as a positive control. 

Induction of proliferation was quantified by counting incorporated [^3^H] thymidine after incubation for 4 days in a humidified atmosphere (37 °C, 5% CO_2_). On the fourth day, 0.5 µCi/well ^3^H-thymidine (ICN, Irvine, USA) was added, suspensions were incubated for 18 h and plates were frozen at −20 °C overnight. Incorporated radioactivity was counted with a Microbeta Trilux 1450 scintillation counter (Wallac, Turku, Finland). Counts per minute were given as corrected counts per minute (ccpm), based on automated counter protocols defining counting parameters. Neutralization of T cell activation was defined as a ≥50% decrease of thymidine incorporation compared to results for negative serum.

### 4.4. Gene Expression Assessed by Quantitative Real-Time PCR

For cytokine gene expression, 5 × 10^6^/mL cells were cultured in flat-bottom tissue culture plates and stimulated after four days with 1 ng/mL wild-type recombinant TSST-1 for 5 h in a humidified atmosphere. For the assessment of neutralization of T cell associated cytokines important for the cytokine storm and inflammatory cytokines, quantitative real time PCR was done for human IL-2, IFNγ, TNFα, IL-6 and HPRT. Phorbol myristate acetate (PMA) and ionomycin were used as positive controls. Human RNA extraction and reverse transcription were performed as described elsewhere [26,31]. Briefly, RNA was extracted from frozen cell pellets using a RNA isolation kit (Roche, France). Extracted RNA was transcribed into cDNA using a reverse transcription kit (Invitrogen, Paisley, UK). Primer design and quantitative real-time PCR were previously described [26,31]. Primer sequences are shown in Table 1.

Upon the addition of KAPA SYBR FAST Supermix (Peqlab, Erlangen, Germany), amplification was done using an ABI Prism 7500-FAST (Applied Biosystems, Vienna, Austria). For the calculation of amplified cDNA copy numbers of target genes, cycles at threshold (CT) values were plotted against logarithmic values of cDNA standard copy numbers on a standard curve. Ultimately, fold induction of mRNA expression was calculated from values normalized using both the expression of the internal standard house-keeping gene HPRT and mean values derived from unstimulated cells of three human donors. More precisely, to calculate the fold induction, we formed Ct values to the 10th of the cytokine genes and those of the housekeeping gene HPRT. Then, we divided the potency of the cytokine gene by that of the HPRT gene. Then, we divided the quotient by the unstimulated cytokine-specific mean value, which resulted in the fold induction. Neutralization was defined as >50% of inhibition of cytokine gene expression.

### 4.5. Neutralizing Antibodies

Neutralizing antibodies against uncontrolled T-cell activation and cytokine gene expression were assessed. Neutralizing antibodies were tested in four semi-logarithmic dilutions (1:100, 1:300, 1:1000 and 1:3000). For the determination of inhibition of ^3^H thymidine incorporation (lymphocyte proliferation) as described above, suspensions of diluted sera and wild type recombinant TSST-1 were incubated for 1 h at 37 °C, added to freshly isolated and cultured cells and incubated for 4 days at 37 °C in a humidified atmosphere.

For testing neutralization of cytokine gene expression (IL-2, IFNγ TNFα and IL6) by quantitative RT-PCR, diluted sera were added to cells, which were isolated and cultured 24 h before at 37 °C in a humidified atmosphere. Upon addition of sera, wild type recombinant TSST-1 was added to stimulate cells and incubated for 5 h at 37 °C in a humidified atmosphere. 

Neutralization was defined as >50% of inhibition of cytokine gene expression; for the calculation, fold induction of each dilution of serum was divided by fold induction of applied antibody negative control serum, incubated in parallel on the same tissue culture plate. The highest dilution with 50% or more inhibition was considered the titer (reference: antibody negative serum).

### 4.6. Statistical Analysis

We compared neutralization antibody titer seroconversion rates between immunized and placebo groups using Fisher’s exact test. Comparisons between screening and immunization rounds with rTSST-1 were done using the paired, two-tailed Wilcoxon test. A *p*-value < 0.05 was considered statistically significant. Data were analyzed with GraphPad Prism 7 (GraphPad Software, San Diego, CA, USA).

## Figures and Tables

**Table 1:** Primer pairs designed for quantitative RT-PCR.

Name	Sequence
HPRT fwd	AGGCCATCACATTGTAGCCC
HPRT rev	GTTGAGAGATCATCTCCACCG
IL-2 fwd	AAACCTCTGGAGGAAGTG
IL-2 rev	GTTCAGAAATTCTACAATGG
IFN fwd	GGCTGTTACTGCCAGGAC
IFN rev	GGAGACAATTTGGCTCTG
TNF fwd	CTGTACCTCATCTACTCCC
TNF rev	GAGAGGAGGTTGACCTTG
IL-6 fwd	AGCCCTGAGAAAGGAGACAT
IL-6 rev	CAAGTCTCCTCATTGAATCC
